# Editorial: RNA helicases: pioneering therapeutic avenues in cancer treatment

**DOI:** 10.3389/fcell.2026.1900371

**Published:** 2026-07-14

**Authors:** Lidia Chellini, Takahiko Murayama

**Affiliations:** 1 Laboratory of Cellular and Molecular Neurobiology, Santa Lucia Foundation (IRCCS), Rome, Italy; 2 Department of Cell Biology, SUNY Downstate Health Sciences University, Brooklyn, NY, United States

**Keywords:** cancer therapy, DEAD/DExH-box, gene expression, R-loop, RNA helicase

## Introduction

The Research Topic entitled *“RNA Helicases: Pioneering Therapeutic Avenues in Cancer Treatment”* explores the emerging role of RNA helicases as central regulators of fundamental cellular processes whose dysregulation contributes to tumor initiation, progression, and therapeutic resistance. Over the last decade, increasing evidence has highlighted the importance of these enzymes not only in RNA metabolism but also in the maintenance of genome stability, cellular stress responses, and immune regulation, positioning them as attractive targets for cancer therapy.

The aim of this Research Topic is to provide new evidence supporting the functional relevance of RNA helicases as cancer-associated regulators while highlighting molecular mechanisms that may be exploited for therapeutic intervention. In addition, the Research Topic discusses emerging strategies to improve treatment efficacy by targeting RNA helicases directly or by exploiting pathways that depend on their activity.

This Research Topic includes four articles, comprising one Original Research article and three Mini-Review articles. Together, these contributions provide a comprehensive overview of the diverse biological functions of RNA helicases, including their roles in RNA processing, R-loop resolution, cellular stress responses, innate immunity, and transcriptional regulation. Collectively, the articles emphasize the remarkable functional versatility of this protein family and its growing relevance in cancer biology.

The Original Research article by Beauchemin et al., entitled *“Inhibiting the RNA helicase DDX3X in Burkitt lymphoma induces oxidative stress and impedes tumor progression in xenografts,”* investigated whether DDX3X, an RNA helicase frequently mutated in Burkitt lymphoma (BL), could serve as a novel therapeutic target for this disease. Using both *in vitro* and *in vivo* experimental models, the authors demonstrated that pharmacological inhibition of DDX3X with RK-33, as well as genetic depletion of the helicase, significantly reduced tumor progression by inducing cellular stress responses and impairing cancer cell survival. Furthermore, a CRISPR-based chemogenomic screen identified the glutathione synthesis pathway as a critical determinant of sensitivity to DDX3X inhibition. Notably, suppression of glutathione production enhanced the cytotoxic effects of RK-33, suggesting a potential combination strategy to improve therapeutic efficacy in BL.

The three Mini-Review articles focus on the biological functions of RNA helicases and their interaction partners, including the histone methyltransferase EHMT2, the fusion oncoprotein PAX3-FOXO1, adenosine deaminase acting on RNA 1 (ADAR1), and several factors involved in R-loop resolution. These reviews highlight the growing evidence supporting RNA helicases as promising therapeutic targets across different cancer types.

The first Mini Review, by Bianconi and Mozzetta, entitled *“Unwinding New Therapeutic Opportunities in Rhabdomyosarcoma: The Role of RNA Helicase DDX5,”* discusses the role of DDX5 as a key oncogenic regulator in rhabdomyosarcoma (RMS), particularly in the aggressive fusion-positive alveolar subtype (Gualtieri et al., 2022). DDX5 promotes tumor growth and survival by stabilizing oncogenic pathways involving EHMT2 and PAX3-FOXO1 (Gualtieri et al., 2022) and by cooperating with YTHDC1 to enhance the biogenesis of cancer-associated circular RNAs (Dattilo et al., 2023). Given its overexpression and functional importance in RMS cells, DDX5 represents an attractive therapeutic target. The review also discusses emerging DDX5 inhibitors, including RX-5902, which may offer new opportunities for targeted treatment approaches in RMS.

The second Mini Review, by Minakuchi et al., entitled *“Orchestrating Innate Immunity Through RNA Editing and Helicase Activity: ADAR1, dsRNA Sensors, and Tumor Immune Evasion,”* examines the role of ADAR1 as a key RNA-editing enzyme that maintains cellular double-stranded RNA (dsRNA) homeostasis. Accumulation of endogenous dsRNA can activate innate immune pathways, leading to inflammatory responses and cell death (Chen and Hur, 2022). ADAR1 prevents inappropriate immune activation by catalyzing adenosine-to-inosine (A-to-I) editing of endogenous dsRNA molecules. The review provides a detailed overview of A-to-I RNA editing and highlights how ADAR1 cooperates with RNA helicases, including DDX3X and DHX9, to regulate dsRNA sensing and immune signaling. These findings have important implications for cancer therapy, particularly in the context of immunomodulatory approaches.

The third Mini Review, by Herrera et al., entitled *“Targeting R-loops: Diverse RNA Helicases in R-loop Resolution and Their Potential as Targets for Cancer Therapy,”* focuses on the critical role of RNA helicases in maintaining genome stability through the resolution of R-loops. The authors summarize evidence demonstrating that defects in R-loop resolution lead to replication stress, DNA damage, transcriptional abnormalities, and genomic instability, all of which are hallmarks of cancer (Niehrs and Luke, 2020; Yang et al., 2023). The review highlights a growing list of RNA helicases involved in R-loop metabolism and discusses how understanding their recruitment mechanisms and substrate specificity may reveal new therapeutic vulnerabilities in cancer cells.

Overall, RNA helicases have emerged as key multifunctional regulators of RNA processing and cellular homeostasis whose activities are frequently altered in cancer. The objective of this Research Topic was to provide an updated perspective on the biological functions of RNA helicases and to evaluate their potential as therapeutic targets. The four contributions published in *Frontiers in Cell and Developmental Biology* significantly advance our understanding of RNA helicase biology and its possible clinical translation.

We anticipate that this Research Topic will be of interest to a broad readership, including basic researchers, translational scientists, and clinicians working in oncology and RNA biology. Nevertheless, these articles address only a fraction of the many unresolved questions surrounding the diverse and context-dependent functions of RNA helicases. Further investigations will be essential to elucidate the molecular mechanisms governing helicase activity, identify predictive biomarkers of response, and develop more selective and effective therapeutic strategies with reduced toxicity. Continued research in this rapidly evolving field is expected to uncover new opportunities for targeting RNA helicases and their associated pathways in cancer treatment.
